# Accelerated Aging Ultraviolet of a PET Nonwoven Geotextile and Thermoanalytical Evaluation

**DOI:** 10.3390/ma15124157

**Published:** 2022-06-11

**Authors:** Yara Barbosa Franco, Clever Aparecido Valentin, Marcelo Kobelnik, Jefferson Lins da Silva, Clovis Augusto Ribeiro, Marta Pereira da Luz

**Affiliations:** 1 São Carlos School of Engineering-EESC, University of São Paulo—USP, São Carlos 13566-590, Brazil; yarabf@usp.br (Y.B.F.); cclever@sc.usp.br (C.A.V.); mkobelnik@gmail.com (M.K.); 2 Departamento de Química Analítica, Universidade Estadual Paulista—UNESP, Araraquara 14801-970, Brazil; clovis.ribeiro@unesp.br; 3 Department of Dam Safety and Technology, Furnas Centrais Elétricas S.A., BR153, km 510, Zona Rural, Aparecida de Goiânia 74923-650, Brazil; martaluz@furnas.com.br; 4 Industrial and Systems Engineering Postgraduate Program (MEPROS), Pontifical Catholic University of Goiás—PUC Goiás, Goiânia 74605-220, Brazil

**Keywords:** polyester, nonwoven geotextile, thermal analysis, physical tests

## Abstract

Nonwoven geotextiles are geosynthetic products that are highly susceptible to ultraviolet degradation because light can reach a large area of the material due to its fiber arrangement. Even with additives, which delay the degradation process, material decomposition still occurs, and therefore the product’s long-term durability can be affected. In this paper, the mechanical and thermal behavior of a commercial nonwoven polyester geotextile subjected to accelerated ultraviolet aging tests were evaluated. The deterioration was evaluated by comparing the physical properties (mass per unit area, thickness, and tensile strength) and thermal behavior (thermogravimetry—TG, thermomechanical analysis—TMA, and differential scanning calorimetry—DSC) before and after exposure times of 500 h and 1000 h. The results showed that the ultraviolet aging tests induced some damage in the polyester fibers, leading to the deterioration of their tensile strength. For 1000 h of exposure, in which the reduction was larger, scanning electron microscopy (SEM) found some superficial disruption of the fibers, indicative of damage. TG and DSC could not capture the effects of UV radiation on polymer degradation, unlike TMA. This latter technique was effective in showing the differences between specimens before and after UV exposure.

## 1. Introduction

Geosynthetics is a relatively recent material for application in engineering work when compared to most construction materials. As a polymeric material, it is subjected to degradation that impacts its long-term durability, which raises the question of its suitability for use in permanent civil engineering systems designed with long service lifetimes. Aging is a major factor impacting geosynthetic service lifetime, acting even before the material installation in civil and environmental engineering works. For this reason, the success of the solution relies on the proper transport and storage of the geosynthetic material. According to specific guidelines, the product package should be intact and have minimal contact with the atmosphere. In the case of open-air storage, the material should be covered with a black polyethylene sheet to ensure adequate protection from ultraviolet (UV) radiation, moisture, and contaminants. However, when contact with these factors is inevitable, it is advisable to discard the outer roll material [1].

Particularly in the case of geotextiles, direct exposure to sunlight can cause severe degradation [2] due to UV radiation. Sunlight covers a wide range of wavelengths from infrared (>700 nm) to ultraviolet (<400 nm), reaching a lower value of around 300 nm related to atmospheric conditions. According to Suits and Hsuan [3], photons of similar or higher energy than the chemical bond strength of the geosynthetic material can lead to the degradation of the polymer properties (physical, mechanical, and chemical) due to the start of a series of reactions that can break the polymer chain. In the case of polyester (PET), the UV degradation causes polymer chain scission, forming a carboxyl group.

Therefore, as it is one of the major factors responsible for polymeric degradation, the influence of ultraviolet radiation (UV) on geosynthetics behavior, focused on in the present paper, is an important research subject in geotechnical engineering, considering that in many applications these materials are installed in an open environment, subject to sunlight exposure [4,5].

To protect the polymers against UV effects, different products are added during material manufacturing, the so-called UV stabilizers. The most common one is carbon black. This is a particulate added to the material’s surface to mechanically protect it by absorbing UV radiation. However, the absorption efficiency depends on the carbon black particle size. Smaller particles show a larger contact surface and thus offer better protection against UV until a limit of 20 nm size, below which no additional gain is obtained [6]. For geotextiles, for example, the particle size of carbon black is typically in the range of 22–25 nm diameter [3].

The study of UV radiation effects in polymer degradation can be conducted under natural sunlight exposure or under accelerated weathering by the emission of wavelengths in the UV spectrum. According to Suits and Hsuan [3], accelerated laboratory weathering methods can give more consistent results as the environment can be controlled. For these tests, UV weathering chambers equipped with lamps are used that irradiate UV light, exposing the geotextile samples to aggressive cycles of UV light, moisture, and temperature [7]. The currently used equipment differs in terms of the lamp that is used as follows: xenon-arc, carbon-arc, and fluorescent-UV lamps. They work similarly, with programmed cycles of condensation and radiation according to test requirements. However, according to Allen [7], the most used lamp worldwide is the fluorescent-UV lamp because it radiates light at the range of the wavelengths that are more damaging for polymers (300–400 nm) and still presents an economical operation.

Previous studies regarding the effects of UV radiation on geotextile behavior have mainly focused on comparing different weathering methods and the evaluation of the degradation by accessing the loss in mechanical properties of the material, such as the tensile strength and strain. Koerner et al. [2], for instance, evaluated seven nonwoven geotextiles under natural sunlight exposure for 12 months and accelerated laboratory tests for 500 h. The authors evaluated the aging effects by means of tensile tests and observed that the polyester geotextiles were less degraded than the polypropylene ones. Besides, their results indicated that none of the accelerated tests modeled field conditions exactly, and therefore, they should be considered an index test rather than a performance one. Carneiro et al. [8] also compared UV-aging behavior of geotextiles in the laboratory and outdoors. For their study, the authors used a polypropylene (PP) geotextile and evaluated the material changes based on physical and mechanical properties and also on scanning electron microscopy (SEM).

Guimarães et al. [9] studied the synergetic effects between creep and weathering for a woven polypropylene geotextile. The material was submitted to outdoor exposure, and the degradation was evaluated by comparing tensile test results, in which the synergy effects were evident. Carneiro and Lopes [10] also evaluated the changes in the mechanical properties (tensile and static puncture tests). They studied four nonwoven polypropylene geotextiles, with a varied amount of stabilizer in their composition, after natural exposure to sunlight during a total period of three years. They found relevant reductions in the mechanical properties of the material. The authors also used SEM photographs to evaluate the damage to the geotextile fibers due to the exposure. Similar studies were conducted by Carneiro et al. [11] with PP nonwoven geotextiles.

The use of thermal analyses to evaluate the durability of nonwoven geotextiles is still developing [4,5,12,13]. Thomas and Verschoor [12], for example, used thermal analyses (differential scanning calorimetry—DSC, thermomechanical analysis—TMA, and dynamical mechanical analysis—DMA) and physical property testing to study the chemical aging of nonwoven polyester geotextiles. Some studies assessed thermal analyses in different applications in other geosynthetics, such as geomembranes used in environmental works [14,15,16,17]. However, despite their potential, these techniques have still not been used to evaluate the effects of UV radiation on nonwoven PET geotextiles, which is the focus of this study.

Therefore, in order to contribute to the study of geotextile durability, the objective of this paper was to evaluate the suitability of three thermoanalytical methods to access the mechanical, thermal, and thermomechanical effects of UV radiation on nonwoven PET geotextiles. The thermoanalytical studies were carried out with thermogravimetric (TG), DSC, and TMA. The density (specific gravity method) and the tensile strength of the samples (dumbbell-shaped test specimens) were evaluated. Additionally, scanning confocal electron microscopy was used to make a qualitative visual analysis of the samples in an attempt to determine possible microstructural changes in the outside surfaces of the fibers of the geosynthetic material due to the degradation process.

## 2. Experimental

One commercial nonwoven polyester geotextile was used in the present study. This type of material was selected because nonwoven geotextiles have a particularly high susceptibility to photo-initiated degradation due to their large surface area [2].

**Figure 1:**
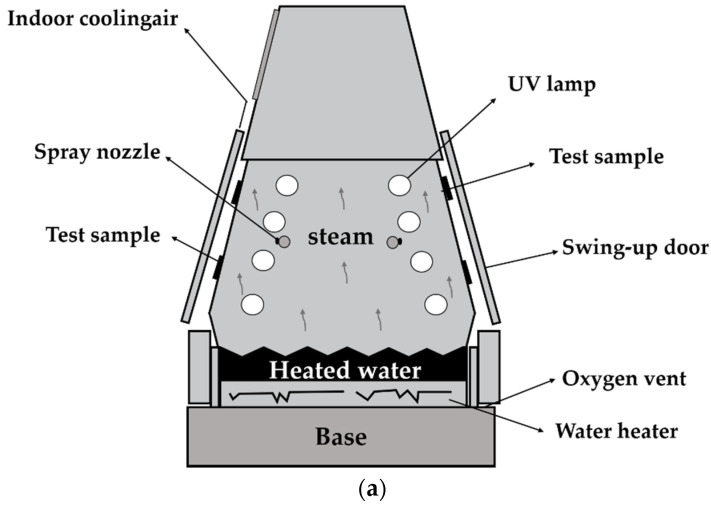

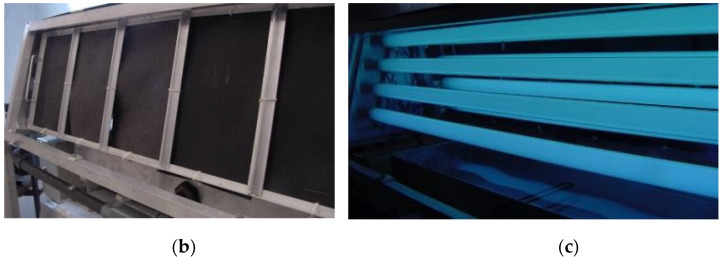
UV weathering chamber: (**a**) schematic of a condensation cycle, (**b**) test sample arrangement, and (**c**) UV lamp detail.

A UV-weathering chamber from Equilam (model EQUV 003, São Paulo, Brazil) with fluorescent UVA-351 lamps was used, programmed to work in cycles of 8 h of UV light at 70 °C followed by 4 h of condensation at 50 °C (Figure 1). Two geotextile samples (20 × 30 cm^2^) were exposed during periods of 500 h and 1000 h, respectively. After exposure, sub-samples were taken from each sample to perform physical, mechanical, and thermal analyses of the aged material. The effects of aging were then evaluated by comparing the results between virgin (reference) and aged samples.

### 2.1. Material Characterization

#### 2.1.1. Physical Properties

The mass per unit area (MPU) [18] was determined by weighing ten geotextile specimens (100 ± 0.1 cm^2^) in characterization tests, and one (600 cm^2^) specimen in exposed PET, in a 3100 g weight capacity laboratory balance (model PB3002-S Mettler Toledo, Zurich, Switzerland), with a resolution of ±0.01 g. The thickness [19] was determined by observing the perpendicular distance that a movable plane was displaced by the geotextile from a parallel surface. A thickness gauge of ±0.001 mm precision and a 56.4 mm diameter presser foot were used to apply a pressure of 2 ± 0.1 kPa for 5 s before taking the measurement.

#### 2.1.2. Mechanical Properties

Tensile tests [20] were performed in an Universal Machine (model DL 3000 EMIC, São José dos Pinhais, Brazil) with pneumatic grips, and a 20 kN load cell. The test method covers strip test procedures to determine tensile strength and specimen elongation. The 50 ± 0.5 mm wide specimens and a speed rate of 300 ± 3 mm/min were used. Each test was carried out with five specimens in the machine’s direction of production.

### 2.2. SEM

SEM was performed with a microscope manufactured (ZEISS SIGMA, Oberkochen, Germany) equipped with an Oxford X-ACT EDS/EDS detector and an electron acceleration voltage of 3.0 kV. Geotextile specimens of 5 × 5 mm randomly removed from the virgin and aged samples were examined. Each specimen was fixed to the sample holder with a silver suspension followed by a deposit of gold to provide the electrical conductivity required for SEM analysis. The analyses were conducted with a voltage of 3.0 kV.

### 2.3. Thermoanalytical Methods

The following three thermoanalytical methods were used in this study to examine the effects of aging on the thermal behavior of nonwoven geotextiles: TG, TMA, and DSC.

TG and TMA before and after aging of the PET nonwoven geotextile were carried out using, respectively, an SDT 2960 and an SDT 2930, both from TA Instruments. In the TG analysis, the samples were evaluated in purge gases of carbonic gas and synthetic air with a flow of 100 mL min^−1^. Sample masses of around 3 mg in an alumina crucible were used with an empty alumina crucible used as a reference. For the kinetic analysis study, the heating rates used were of 10, 15, 20, and 30 °C·min^−1^. The kinetic evaluation was taken from the first derivative curves of each heating rate. Activation energies were calculated with the following three isoconversion methods [14]: the Friedman, the Flynn–Wall–Ozawa, and the Capela/Ribeiro method. The TMA analysis, in turn, was carried out with samples of 5 mm × 5 mm. The experimental conditions were as follows: temperature range from 20 °C to 200 °C and a heating rate of 5 °C·min^−1^.

DSC analyses were carried out in a temperature range from 25 °C to 260 °C and from 200 °C to 25 °C with a heating rate of 20 °C·min^−1^ under nitrogen purge gas with a flow of 50 mL min^−1^. The measurements were obtained with the DSC1 Star^e^ (Mettler Toledo, Zurich, Switzerland), using samples with a mass near 2 mg.

## 3. Results and Discussion

### 3.1. SEM

**Figure 2:**
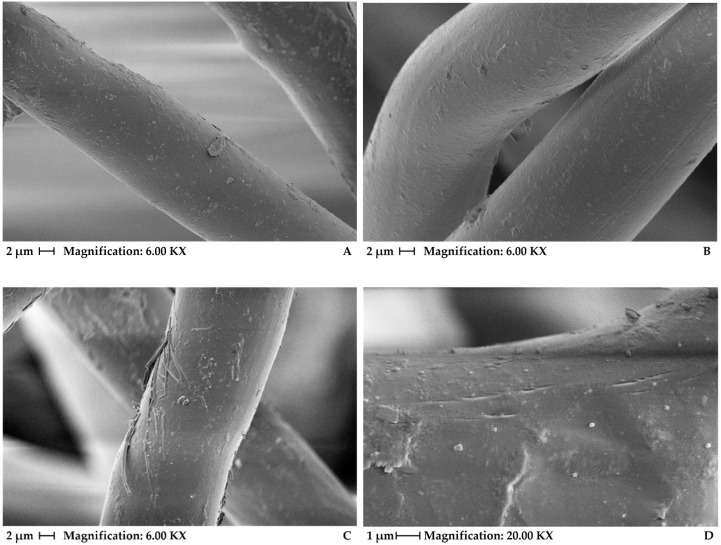
Scanning electronic microscopy: (**A**) virgin PET, (**B**) PET 500 h, (**C**,**D**) PET 1000 h.

Figure 2 shows SEM analyses for virgin PET samples and for PET samples after 500 h and 1000 h of accelerated aging. As seen in Figure 2a, virgin PET samples show the integrity of the material before exposure, in which no rupture of the fibers is observed. For the sample exposed to UV radiation for 500 h (Figure 2b), the fibers did not show a significant change, indicating that this exposure time was insufficient to cause fiber damage. However, for the time of 1000 h, a superficial disruption of the fibers can be seen (Figure 2c). Additionally, in Figure 2d, the presence of longitudinal cracks can be observed, although no transverse cracks are perceived, which is usually observed for degraded PP fibers [8,10,21]. Therefore, for the present study, the number of stabilizers in the commercial PET geotextiles used was sufficient to protect the material for 500 h of accelerated weathering but not for 1000 h of exposure.

A previous study by Carneiro and Lopes [10], using SEM, showed extensive damage to PP geotextiles due to natural exposure of 12 months, with many cracked and broken fibers. However, despite the authors’ having studied geotextiles with different amounts of stabilizers in their composition, they only evaluated the damage with the SEM technique for the unstabilized PP geotextiles. Without any stabilizer, the geotextile is highly susceptible to UV degradation, which explains the damage captured by SEM in their study. Carneiro et al. [8], in turn, used SEM analyses to show that accelerated UV tests induced damage in PP fibers in the function of UV radiant energy. The cracks were observed only in the transverse direction of the fibers. In this case, the geotextile contained 0.4% of the mass of the UV stabilizer Chimassorb 944.

**Figure 3:**
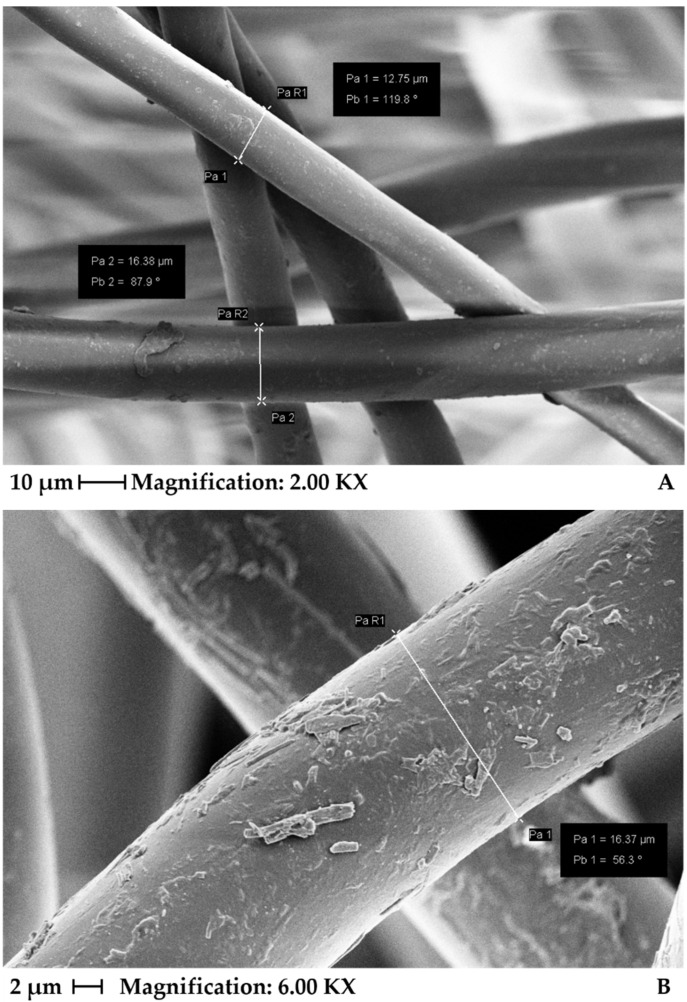
Scanning electronic microscopy indicating some fiber diameters of virgin PET (**A**) and PET 1000 h (**B**).

Figure 3 shows SEM analysis in detail of some fiber diameters, in which there are no visible changes in the fibers due to exposure. As seen for the PET fiber, the diameters are between 12 and 16 µm, while for the fiber exposed for 1000 h, a value of 16.37 µm was found.

### 3.2. TG and Kinetic Evaluations

**Figure 4:**
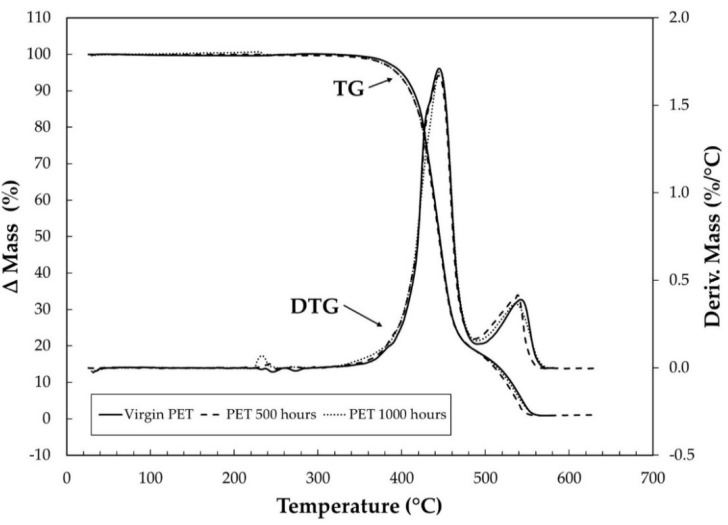
TG/DTG curves of virgin PET, PET 500 h, and PET 1000 h under heating rates of 20 °C·min^−1^ under synthetic air purge gas with flow of 100 mL min^−1^ in α-alumina crucible and sample masses around 3 mg.

**Figure 5:**
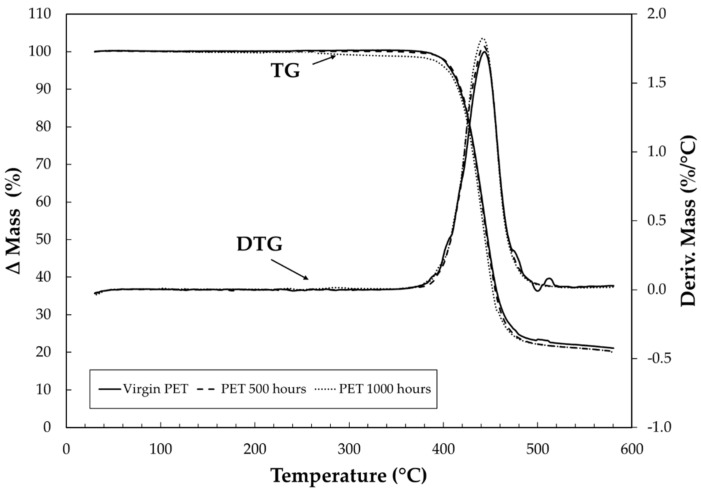
TG/DTG curves of virgin PET, PET 500 h, and PET 1000 h under heating rates of 20 °C·min^−1^ under purge gas of carbonic gas with flow of 100 mL min^−1^ in α-alumina crucible and sample masses around 3 mg.

TG curves were obtained aiming to determine the thermal stability, as well as the influence of UV light on the thermal properties of PET. Having selected TG curves under 20 °C of virgin PET, 500 h and 1000 h of aging are shown in Figure 4 and Figure 5, under purge gases of synthetic air and carbonic gas, respectively. Observing the TG curves in synthetic air (Figure 4), it can be seen that the samples present good thermostability because no significant mass loss occurred until 325 °C. Concerning the effect of the different conditions of analysis (synthetic air and carbonic gas), it was found that the first thermal decomposition stage from TG curves are very similar, with a small difference among them, which is better seen only in derivative thermogravimetry (DTG) curves, for the sample of 1000 h of aging. In the second stage of thermal decomposition, the TG and DTG curves had different displacements for each sample. The temperature for maximum mass loss was 560 °C, and the residual amount of the carbonaceous material was virgin PET with 1.07 %, PET 500 h with 0.92 %, and PET 1000 h with 1.02 %.

**Figure 6:**
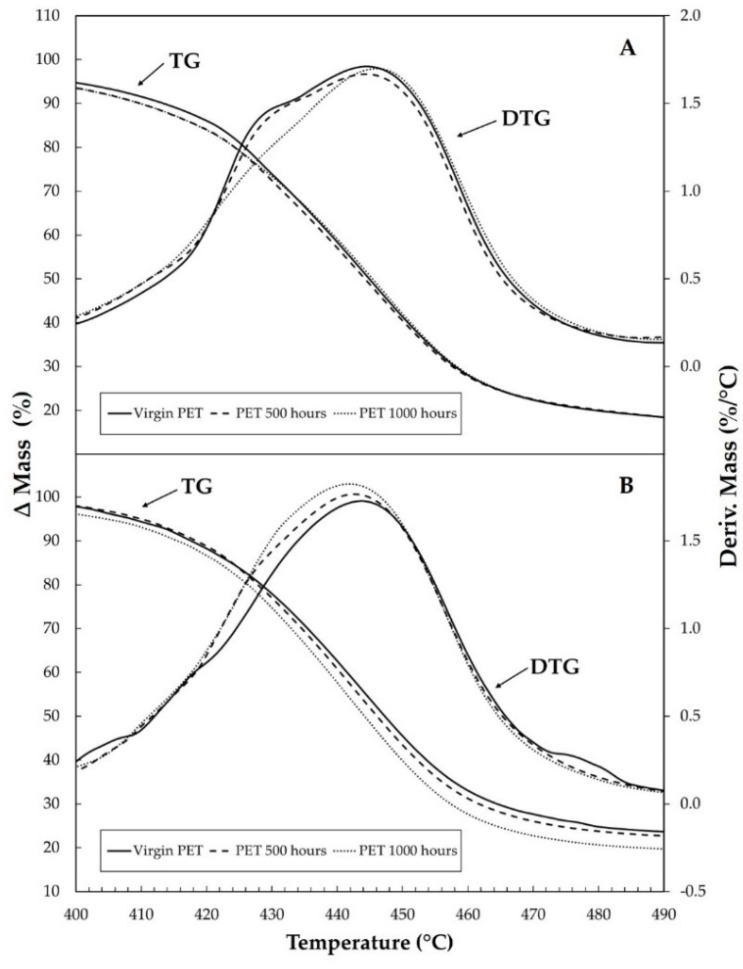
Details of TG/DTG curves of virgin PET, PET 500 h, and PET 1000 h under heating rates of 20 °C·min^−1^ with flow of 100 mL min^−1^ in α-alumina crucible: (**A**) under purge gas of synthetic air and (**B**) with purge gas of carbonic gas.

Figure 5 shows that for the TG/DTG curves under carbonic gas, the thermal decomposition occurs after 341 °C in only one stage, and once again, the curves are very similar. However, in DTG curves, the displacement is very visible. These behaviors can be observed in Figure 6a,b, showing details of the TG and DTG curves in the range of temperature between 400 and 490 °C for purge gases of synthetic air and carbonic gas. The total mass losses were virgin PET with 79.96%, PET 500 h with 79.20%, and PET 1000 h with 81.05%. As can be seen, the detailed curves allow better identification of the displacement and facilitate the interpretation of the results, showing the small effect of UV light on the TG/DTG curve behavior of the samples.

**Figure 7:**
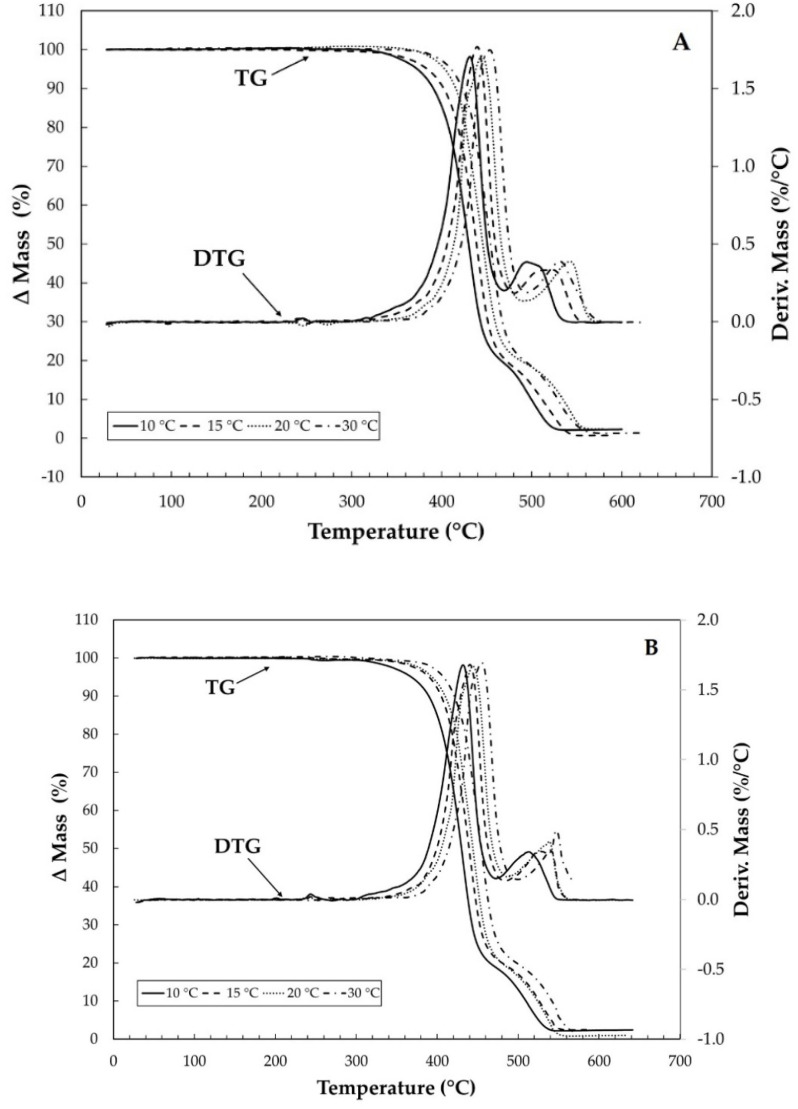

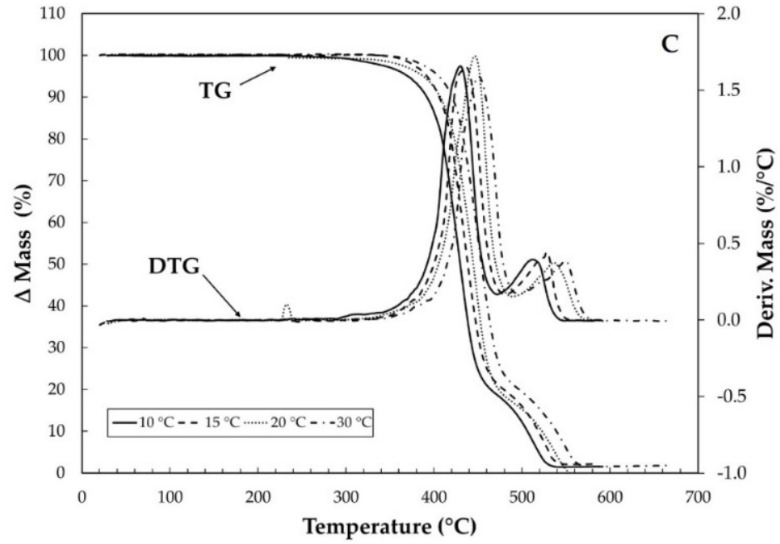
TG/DTG curves of virgin PET (**A**), PET 500 h (**B**), and PET 1000 h (**C**) with heating rates of 10, 15, 20, and 30 °C·min^−1^ under synthetic air gas purge with flow of 100 mL min^−1^ in α-alumina crucible.

**Figure 8:**
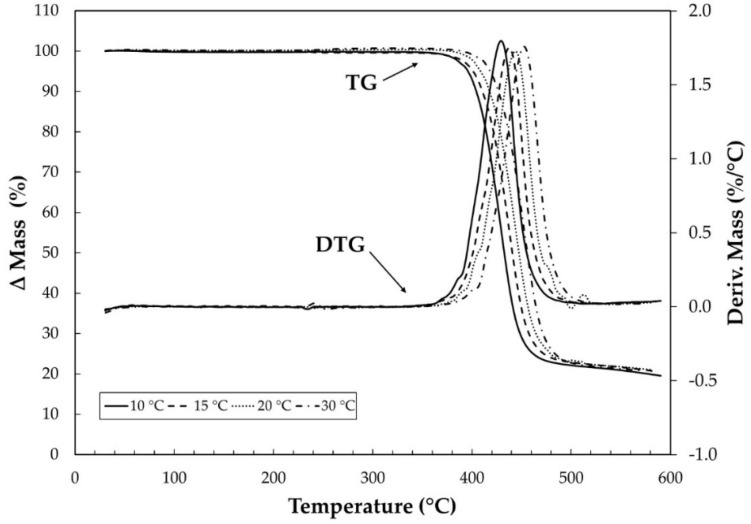
TG/DTG curves of virgin PET with heating rates of 10, 15, 20, and 30 °C·min^−1^ under carbonic gas with flow of 100 mL min^−1^ in α-alumina crucible.

Figure 7 shows the overlays of the TG/DTG curves in synthetic air at different heating rates for the virgin PET samples at 500 h and 1000 h of aging. Figure 8, in turn, shows only the results for virgin PET under carbonic gas, as the other samples (500 h and 1000 h) showed similar behavior. For the study of the activation energy with the curves in synthetic air, only the first stage of the thermal decomposition was considered. In addition, for the analysis of synthetic air for the virgin specimen (Figure 7a), it can be observed that the second stage of thermal decomposition is at 20 °C·min^−1^ superimposes the analysis at 30 °C·min^−1^, while for the analysis of the specimen of 500 h (Figure 7b), the curves for heating rates of 10 and 20 °C·min^−1^ overlap. Only for the sample of 1000 h, there was no overlap of the curves in the second stage of thermal decomposition, as seen in Figure 7c.

**Figure 9:**
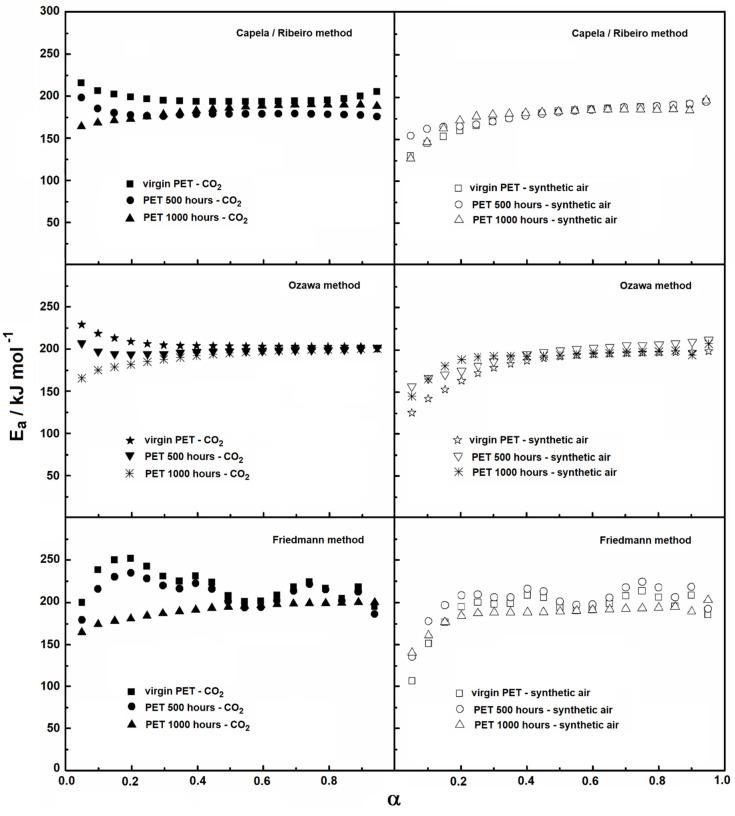
Activation energy versus conversion degree (α) from three isoconversional methods.

The temperature intervals used for the kinetic evaluation are shown in Table 1, while the mean values of activation energy and the coefficients of variation are shown in Table 2. The dependence of activation energy on the degree of conversion (α) for the samples is shown in Figure 9. The variation observed in the activation energy under purge gases of carbonic gas and synthetic air suggests that the process is kinetically variable with all methods used. The evaluation from the Capela/Ribeiro and Ozawa methods indicates that the degradation kinetics is governed by unique processes at the initial and final stages, while the Friedmann method had greater variations throughout the range of conversion degree for virgin PET samples and for samples after aging of 500 h.

For all analysis methods under synthetic air, the first region of the graph (Figure 9) corresponds to values of *α* up to almost 0.2, after which the activation energy presents an increase and remains almost constant throughout the whole conversion range. On the other hand, under carbonic gas and for virgin PET samples and PET samples submitted to 500 h of aging, different kinetic behaviors can be observed, considering the Capela/Ribeiro and Ozawa methods, in which the initial values present a decrease. For the sample of 1000 h of aging, in contrast, an increase in activation energy was observed to a value of conversion degree equal to 0.2. These variations of the activation energy are linked to the complexity of the thermal degradation. Thus, only considering the knowledge of the complexity of the reactions and their respective reaction mechanisms is it possible to make the connection with the activation energy adjustments to obtain reaction mechanisms, which is not the scope of this paper.

### 3.3. DSC and TMA

**Figure 10:**
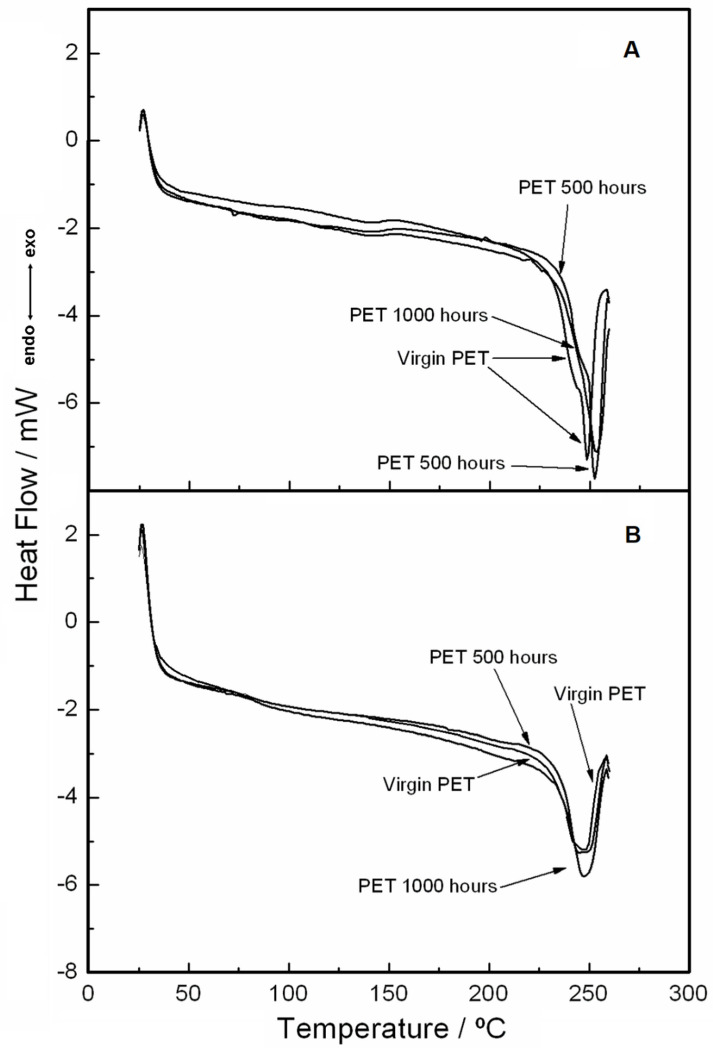
DSC curves with a heating rate of 20 °C·min^−1^ under nitrogen gas purge with a flow of 50 mL min^−1^ in an aluminum crucible with sample masses around 2 mg: (**A**) first heating and (**B**) second heating.

**Figure 11:**
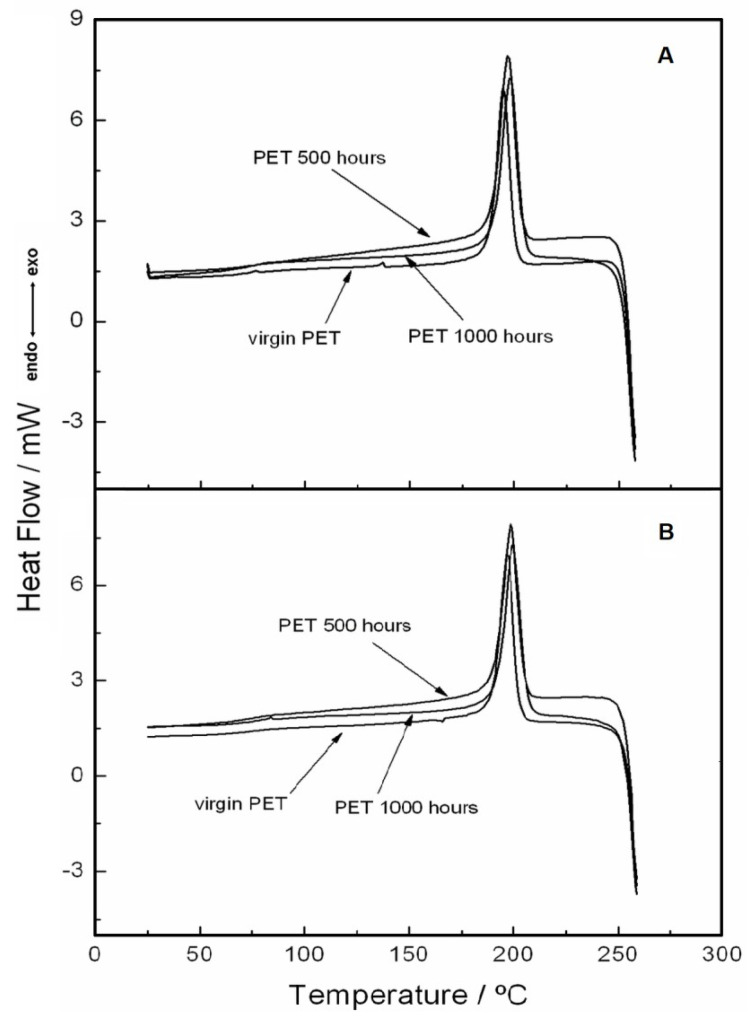
DSC curves with a cooling rate of 20 °C·min^−1^ under a nitrogen gas purge with a flow of 50 mL min^−1^ in an aluminum crucible with sample masses around 2 mg: (**A**) first cooling and (**B**) second cooling.

Figure 10 and Figure 11 show the DSC curves obtained under two heating and two cooling conditions, respectively. The second heating was performed after the first heating and cooling, both with a heating rate of 20 °C·min^−1^.

In Figure 10 A,B, DSC curves carried out under the two heating stages are shown. In Figure 10A, related to the first heating, two different melting points (very close to each other) are illustrated (245 °C for virgin PET, 248 °C for 500 h, and 249 °C for 1000 h). The small displacement of the melting point between the virgin sample and the samples exposed to UV light shows that the effect caused by light was not sufficient to significantly change the thermal behavior of the material. In the second heating (Figure 10B), there was an enlargement of the DSC curves (melting points are coincident), which was attributed to the relaxation of the PET fibers due to the better homogeneity caused by the first heating. For the first and second cooling stages (Figure 11 A,B, respectively), it can be observed that the crystallization peaks are very close to each other. This was attributed to the homogenization effect of the sample during melting, and thus, even with the second cooling, the sample remained with the same characteristics.

**Figure 12:**
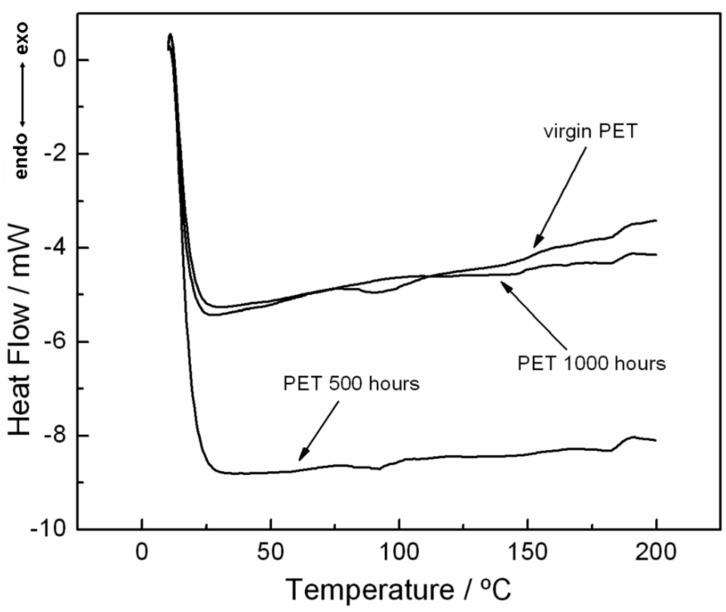
Analysis of glass transition from DSC curves with a heating rate of 30 °C·min^−1^ under nitrogen gas purge with flow of 50 mL min^−1^ in an aluminum crucible.

In addition to the melting and crystallization behavior, another important characteristic of PET materials is the glass transition, which has been studied by many authors. De Clerck et al. [22], for instance, studied fibers and microfibers of PET, and the DSC curves indicated that pure PET had a glass transition between 70 and 80 °C. However, after studying the microfibers, the glass transition disappeared when compared to bulk PET. According to the authors, this fact was attributed to the morphology of the materials studied; that is, the individual fibers are different from the bulk material. As seen in the DSC curves of Figure 10, the glass transition is not well defined, which is attributed to the characteristics of the material used, as follows: As the nonwoven geotextile comprises interlaced PET fibers, the movement of the fibers is not enough to be detected by the thermocouple of the calorimeter. Figure 12 shows an analysis made with a heating rate of 30 °C·min^−1^, which was performed to determine the glass transition. In this figure, it can be observed that the virgin PET curve shows a displacement in the curve between 70 and 107 °C, which was attributed to the glass transition, whereas for the analysis of the sample of 500 h, this reaction occurs at a lower intensity, in which the displacement occurs between 75 and 100 °C. However, for the analysis of 1000 h, there is no displacement in the DSC curve. The lack of video transition at the same interval was attributed to the effect of exposing the sample at 1000 h.

**Figure 13:**
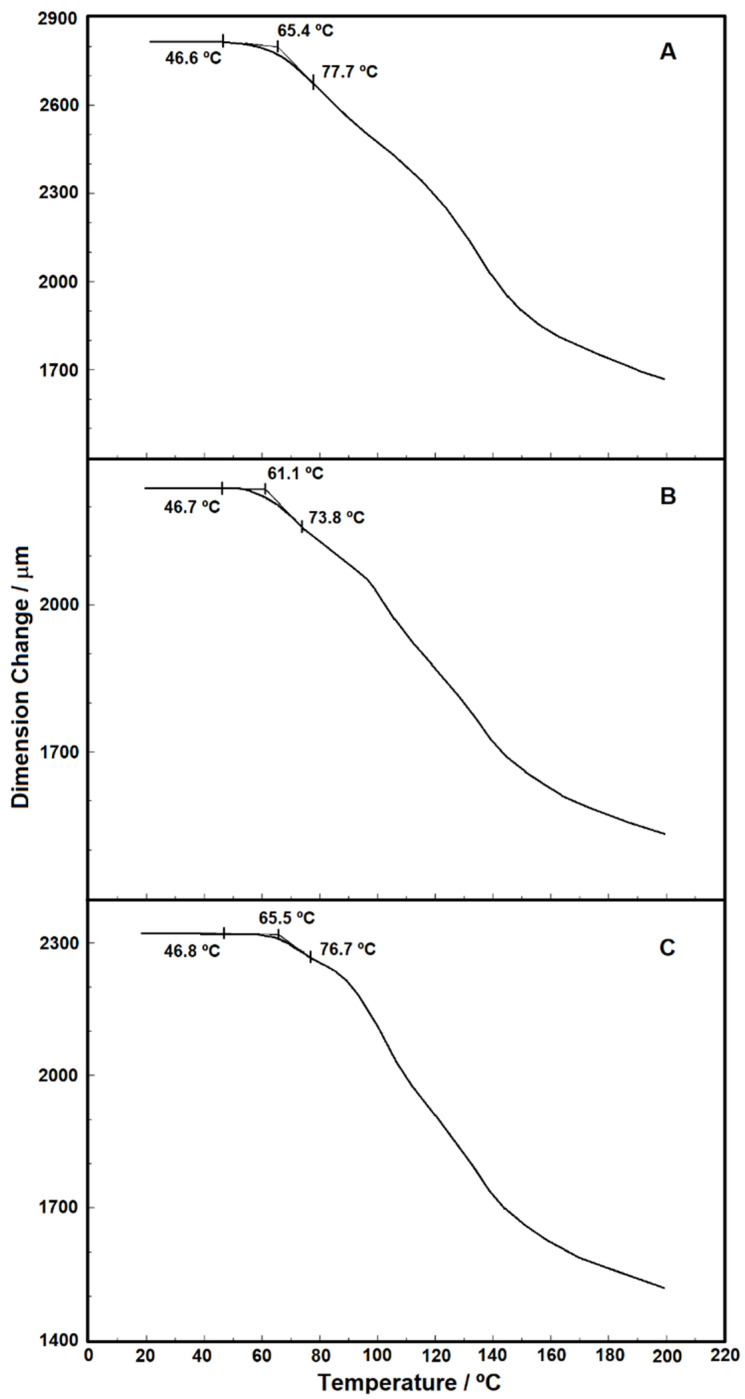
TMA curves of glass transition of virgin PET (**A**), PET 500 h (**B**), and PET 1000 h (**C**).

**Figure 14:**
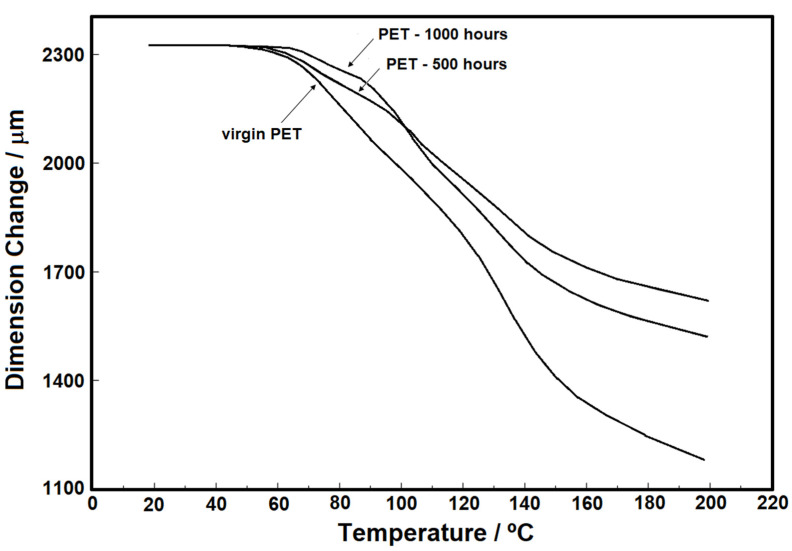
TMA curves of virgin PET, PET 500 h, and PET 1000 h overlapped.

In addition to the thermogravimetry and calorimetry studies, Figure 13 and Figure 14 show the individuals and overlapped curves obtained using the TMA technique for the samples under nitrogen purge gas in the range from 20 to 200 °C without force application. The values of dimension change (µm) in Figure 14 were normalized in order to allow the comparison of the analyses, while in Figure 13A–C, the values presented are the true values for each sample studied. In the literature, studies with thermomechanical analysis (TMA) are scarce, probably due to the greater use of DMA. However, TMA is an important technique in the field of thermal analysis, which allows the evaluation of the dimensional properties of a sample during heating or cooling, or even under isothermal conditions. Besides, the possibility of applying load to the sample allows this technique to be used to assess some important properties, such as the glass transition, melting temperatures, stress relief effects at glass transition, coefficients of thermal expansion (CTE), etc., [23,24].

In Figure 13A–C, the individual analysis for each sample (virgin, 500 h, and 1000 h) is shown, and the respective onset temperatures of glass transition. These results show that, regardless of the condition considered, the samples are thermally stable up to about 50 °C. Nevertheless, the observed thermal behavior shows that the samples are susceptible to heat, i.e., suggestively, this material should not be stored at temperatures above 50 °C. The importance of this result is linked to the application of this type of material in tropical countries, such as Brazil, where the areas of insolation are constant and storage in sheds without air conditioning can easily raise the temperature above 50°C, thus causing a change in material characteristics. Figure 14 shows the overlapping of the TMA curves, in which the behavior of the thickness decrease for the three samples can be compared. The virgin PET sample showed a thickness decrease greater than that of the samples with 500 and 1000 h of aging. In addition, the sample of 1000 h with the longest exposure time to UV radiation showed the smallest thickness decrease.

### 3.4. Physical Evaluations

Table 3 shows the results from the physical evaluations undertaken for samples of virgin PET and PET exposed to UV light for 500 h and 1000 h.

MPU and thickness measurements show no significant change after exposure, considering the typical variability of this type of material. However, tensile strength values tend to decrease (Table 3). After 500 h and 100 h of accelerated aging, the geotextile had a retained tensile strength (at peak) of 86.8% and 81.5%, respectively. These values show that some degree of degradation has occurred, but it was not very large. This correlates well with SEM analyses (Figure 2), in which the fibers did not show a significant change for 500 h of UV exposure and only a superficial disruption of the fibers after 1000 h of aging. With larger reductions in tensile strength, it is expected to see significant damage in the fibers by SEM analyses, as shown by Carneiro et al. [11,21]. For the other elongations evaluated (5%, 10%, 15%, and 20%), a deterioration in tensile strength was also observed. Elongation at maximum load did not change significantly.

It is worth mentioning that, given the variability of the geotextile manufacturing process, it is difficult to isolate the variation in specimen mass, thickness, and tensile properties due to the degradation process only, and thus, one should be cautious when comparing these values.

## 4. Conclusions

The results presented have shown that the PET nonwoven geotextiles studied herein are susceptible to degradation by UV radiation and, therefore, they should be properly protected during storage.

Regarding the thermal analyses conducted, the following was shown:

- The TG analysis was not able to capture the effects of UV radiation on the samples of PET nonwoven geotextiles. This observation is attributed to the melting of the sample during heating, as follows: when melted, PET becomes more homogeneous and, consequently, the effects caused by UV deterioration are reduced;
- The same conclusion is valid for the DSC analyses, in which the curves for different samples showed very similar thermal behavior. Therefore, the TG and DSC techniques are not the most appropriate ones for the analysis of the effects of UV radiation on PET nonwoven geotextiles;
- Nevertheless, the TMA evaluation was satisfactory as this technique evaluates the physical behavior of the sample. Indeed, the TMA results showed the differences between the samples (virgin, 500 h, and 1000 h of aging) more precisely than the TG and DSC techniques.

Concerning the physical evaluations, the accelerated laboratory UV-aging tests did not significantly change the physical properties of the PET geotextile (mass per unit area and thickness). However, tensile strength had a small decrease, larger for 1000 h of exposure to UV radiation, which is an indication of some level of degradation. SEM analyses corroborated this finding, showing a superficial disruption of the fibers after 1000 h of aging. Therefore, the use of thermoanalytical methods to evaluate the influence of UV radiation on the properties of geotextiles is recommended.

## Figures and Tables

**Table 1:** Temperature intervals (°C) of the first stage of thermal decomposition.

Compound	10 °C·min^−1^	15 °C·min^−1^	20 °C·min^−1^	30 °C·min^−1^
Virgin PET (synthetic air) Virgin PET (carbonic gas)	377–451 °C 395–457 °C	392–461 °C 401–466 °C	400–458 °C 406–475 °C	419–476 °C 413–480 °C
PET 500 h (synthetic air)	385–453 °C	398–459 °C	403–467 °C	411–477 °C
PET 500 h (carbonic gas) PET 1000 h (synthetic air)	395–457 °C 384–453 °C	398–464 °C 391–462 °C	404–470 °C 400–469 °C	411–482 °C 412–477 °C
PET 1000 h (synthetic air)	388–455 °C	398–463 °C	415–470 °C	413–479 °C

**Table 2:** Activation energy values using the Flynn–Wall–Ozawa, the Capela/Ribeiro and the Friedmann methods. The data were obtained with the arithmetic mean (r is the correlation value and CV is the coefficient of variation).

Compound and Purge Gas	Capela and Ribeiro Method E_a_/kJ mol^−1^ ± r (CV)	Ozawa Method E_a_/kJ mol^−1^ ± r (CV)	Friedman Method E_a_/kJ mol^−1^ ± r (CV)
Virgin PET (synthetic air)	174.55 ± 0.1 (0.99669)	177.71 ± 0.11 (0.99632)	191.24 ± 0.12 (0.9778)
Virgin PET (carbonic gas)	197.55 ± 0.02 (0.99771)	206.23 ± 0.03 (0.99866)	220.66 ± 0.07 (0.98491)
PET 500 h (synthetic air)	177.48 ± 0.06 (0.99907)	188.85 ± 0.08 (0.99426)	202.47 ± 0.09 (0.98020)
PET 500 h (carbonic gas)	179.42 ± 0.02 (0.99537)	197.63 ± 0.01 (0.99170)	211.13 ± 0.07 (0.97288)
PET 1000 h (synthetic air)	175.82 ± 0.09 (0.99618)	185.99 ± 0.07 (0.98971)	199.96 ± 0.1 (0.97869)
PET 1000 h (carbonic gas)	182.27 ± 0.04 (0.99963)	191.27 ± 0.05 (0.99963)	336.32 ± 0.06 (0.97821)

**Table 3:** Physical properties of the geotextile before and after UV-aging tests.

Sample	MPU (g m^−2^)	Thickness (mm)	Tensile Strength (kN m^−1^)	Elongation at Maximum Load (%)	Load at a Specific Elongation (kN m^−1^)			
					5%	10%	15%	20%
Virgin PET	421.75	3.12 (5.24%)	14.68 (4.42%)	74.32 (9.10%)	1.22 (19.45%)	1.91 (17.62%)	2.58 (18.97%)	3.39 (17.76%)
PET 500 h	421.38	2.98 (3.24%)	12.74 (4.47%)	72.07 (6.94%)	1.10 (17.51%)	1.79 (17.48%)	2.45 (17.72%)	3.17 (17.76%)
PET 1000 h	418.63	3.20 (4.14%)	11.96 (6.08%)	75.12 (4.55%)	0.98 (12.05%)	1.70 (13.14%)	2.35 (13.00%)	3.02 (13.00%)

(Coefficient of variation).

## Data Availability

The data presented in this study are available on request from the corresponding author.

## References

[1] Vertematti J.C. (2004). Manual Brasileiro de Geossintéticos.

[2] Koerner G.R., Hsuan G.Y., Koerner R.M. (1998). Photo-Initiated Degradation of Geotextiles. J Geotech. Geoenvironmental Eng..

[3] Suits L.D., Hsuan Y.G. (2003). Assessing the photo-degradation of geosynthetics by outdoor exposure and laboratory weatherometer. Geotext. Geomembr..

[4] Valentin C.A., Kobelnik M., Franco Y.B., Lavoie F.L., Lins da Silva J., Luz M.P. (2021). Study of the ultraviolet effect and thermal analysis on polypropylene nonwoven geotextile. Materials.

[5] Ardila M.A.A., Pedroso G.O.M., Kobelnik M., Valentin C.A., Luz M.P., Lins da Silva J. (2021). Evaluating the degradation of a nonwoven polypropylene geotextile exposed to natural weathering for three years. Int. J. Geosynth. Ground Eng..

[6] CABOT (1990). Carbon Blacks for Protection of Plastics Exposed to Ultraviolet Light.

[7] Allen S.R., Koerner R.M. (2016). Geotextile Durability. Geotextiles. From Design to Applications.

[8] Carneiro J.R., Almeida P.J., Lopes M.d.L. (2019). Evaluation of the Resistance of a Polypropylene Geotextile Against Ultraviolet Radiation. Microsc. Microanal..

[9] Guimarães M.G.A., de Mattos Vidal D., de Carvalho Urashima D., Castro C.A.C. (2017). Degradation of polypropylene woven geotextile: Tensile creep and weathering. Geosynth. Int..

[10] Carneiro J.R., Lopes M.L. (2017). Natural weathering of polypropylene geotextiles treated with different chemical stabilisers. Geosynth. Int..

[11] Carneiro J.R., Almeida P.J., Lopes M.D.L. (2011). Accelerated weathering of polypropylene geotextiles. Sci. Eng. Compos. Mater..

[12] Thomas R., Verschoor K., Peggs I. (1990). Thermal Analysis of Nonwoven Polyester Geotextiles. Geosynthetics: Microstructure and Performance.

[13] Ardila M.A.A., Santos Junior R.D., Kobelnik M., Valentin C.A., Schliewe M.S., Coelho A.T., Lins da Silva J., Luz M.P. (2021). Semi-Rigid Erosion Control Techniques with Geotextiles Applied to Reservoir Margins in Hydroelectric Power Plants, Brazil. Water.

[14] Valentin C.A., Da Silva J.L., Kobelnik M., Ribeiro C.A. (2019). Thermoanalytical and dynamic mechanical analysis of commercial geomembranes used for fluid retention of leaching in sanitary landfills. J. Therm. Anal. Calorim..

[15] Lavoie F.L., Kobelnik M., Valentin C.A., Da Silva J.L. (2020). Durability of HDPE geomembranes: An Overview. Química Nova.

[16] Lavoie F., Valentin C.A., Kobelnik M., Lins da Silva J., Lopes M.L.C. (2020). HDPE Geomembranes for environmental protection: Two case studies. Sustainability.

[17] Lavoie F.L., Kobelnik M., Valentin C.A., Tirelli E.F.S., Lopes M.L.C., Lins da Silva J. (2021). Laboratory study of the ultraviolet radiation effect on an HDPE geomembrane. Membranes.

[18] (2003). Standard Test Method for Measuring Mass per Unit Area of Geotextiles.

[19] (2006). Standard Test Method for Measuring the Nominal Thickness of Geosynthetics.

[20] (2008). Standard Test Method for Breaking Strength and Elongation of Textile Fabrics (Strip Method).

[21] Tisinger L., Peggs I., Dudzik B., Winfree J., Carraher C. (2009). Microstructural Analysis of a Polypropylene Geotextile After Long-Term Outdoor Exposure. Geosynthetic Testing for Waste Containment Applications.

[22] De Clerck K., Rahier H., Van Mele B., Kiekens P. (2003). Thermal properties relevant to the processing of PET fibers. J Appl. Polym. Sci..

[23] Brown M.E. (1988). Introduction to Thermal Analysis: Techniques and Applications.

[24] Wingard C.D. (1997). Use of thermal analysis techniques for examining blow-molded pet bottle specimens. J. Therm. Anal. Calorim..

